# Lymphatic Mapping to the Inguinal Region in Patients with Lower Extremity Melanoma Undergoing Radical Inguinal Lymph Node Dissection: A Retrospective Cohort Study

**DOI:** 10.3390/cancers18193184

**Published:** 2026-10-02

**Authors:** Zhichao Tan, Yue Sun, Yunxiao Zhang, Zhengfu Fan, Chujie Bai, Shu Li, Tian Gao, Lu Zhang, Ruifeng Xue, Xinyu Wang, Mengmeng Liu, Hairui Wang, Jiayong Liu

**Affiliations:** 1 Key Laboratory of Carcinogenesis and Translational Research (Ministry of Education/Beijing), Department of Bone and Soft Tissue Tumor, Peking University Cancer Hospital & Institute, 100142 Beijing, China; zhichaotan@pku.edu.cn (Z.T.); zhengfufan@126.com (Z.F.); baichujie@163.com (C.B.); lishu_doctor@163.com (S.L.); gaotianauagcu@163.com (T.G.); xue_ruifeng@126.com (R.X.); wxy_196@163.com (X.W.); mengmeng_liu1125@163.com (M.L.); 2 Department of Bone and Soft Tissue Oncology, Peking University Cancer Hospital, Inner Mongolia Campus, 010010 Hohhot, China; 20190118@immu.edu.cn; 3 Affiliated Cancer Hospital of Inner Mongolia Medical University, 010010 Hohhot, China; 4 Key Laboratory of Carcinogenesis and Translational Research (Ministry of Education/Beijing), Department of Anesthesiology, Peking University Cancer Hospital & Institute, 100142 Beijing, China; zhangyunxiao@pku.edu.cn

**Keywords:** inguinal lymph node dissection, melanoma, distribution, lymphatic mapping

## Abstract

**Simple Summary:**

Radical removal of lymph nodes in the groin can cause wound problems and leg swelling. The optimal extent of surgery for patients with clinically involved groin nodes remains uncertain. We examined the locations of removed and cancer-involved lymph nodes in 58 patients with melanoma of the lower limb. Cancer-involved nodes were concentrated in the lower part of the groin, including 19 of 20 patients with only one involved node. Other groin regions were more often involved in patients with a greater number of cancer-involved nodes. This small retrospective study provides preliminary evidence about where involved nodes are found, but does not establish the order in which cancer spreads or show that less extensive surgery is safe. The findings support further studies evaluating cancer control and complications before changing the extent of groin surgery.

**Abstract:**

Background/Objectives: The anatomical distribution of inguinal lymph node (ILN) metastases may inform research on the extent of therapeutic dissection for lower-limb melanoma. Methods: Fifty-eight patients undergoing radical inguinal lymph node dissection (rILND) were retrospectively evaluated using five anatomical zones. Zone-specific node counts were reaggregated, and exploratory binary logistic regression assessed multiple-zone involvement. Results: The cohort included 48 clinically node-positive and 10 sentinel lymph node biopsy (SLNB)-positive patients. Among 816 mapped ILNs, 149 were positive. Zone I contained 102/149 positive nodes (68.5%); within-zone positivity was 102/303 (33.7%) in zone I, 27/243 (11.1%) in zone II, 13/190 (6.8%) in zone III, 3/39 (7.7%) in zone IV, and 4/41 (9.8%) in zone V. Five patients had no positive mapped ILNs. Zone I contained the only positive node in 19/20 patients (95.0%) and was the sole involved zone in 32/33 patients with single-zone involvement (97.0%). Twenty patients had multiple-zone involvement. In a two-variable complete-case model (50 patients; 18 events), ≥3 positive ILNs remained associated with multiple-zone involvement (adjusted odds ratio, 34.80; 95% confidence interval, 5.88–206.08), whereas extranodal extension did not. Conclusions: This small, selected cohort provides preliminary pathological evidence of preferential inferior-zone involvement. It does not establish sequential spread or the oncological safety of reduced rILND. External and prospective validation are required.

## 1. Introduction

Malignant melanoma (MM) frequently involves regional lymph nodes. Sentinel lymph node biopsy (SLNB) positivity varies with patient selection and primary tumor characteristics [1,2,3]. For SLNB-positive patients without clinically evident residual nodal disease, MSLT-II and DeCOG-SLT demonstrated no survival benefit from routine completion lymph node dissection (CLND) compared with observation [4,5]. Contemporary management favors active nodal surveillance, including nodal ultrasonography, rather than routine CLND in this setting [6]. Clinically detectable nodal disease is a distinct setting in which therapeutic lymph node dissection and systemic therapy are considered within multidisciplinary care; neoadjuvant immunotherapy is now an option for resectable macroscopic stage III disease [7]. Groin dissection can cause wound dehiscence, infection, seroma, sensory disturbance, and lymphedema. Melanoma-specific studies document substantial morbidity after inguinal surgery [8,9,10,11]. Reported rates vary with surgical extent, definitions, and follow-up; pooled estimates across malignancies should not be interpreted as melanoma-specific risks [12].

Efforts have been made to minimize the boundaries of rILND. Daseler et al. introduced a traditional method of dividing ILNs into five zones based on cadaver dissection. However, this approach did not delineate the distribution patterns of ILNs, nor could it be directly applied to specific tumor types, such as melanoma or penile cancer (PC). In PC, Catalona first proposed a limited ILND within superomedial regions [13]. Later, D’Ancona et al. implemented modified ILND in a larger cohort of PC patients, achieving a low nodal recurrence rate (5.5%) over two years, alongside a significantly reduced complication rate compared to rILND (38.9% vs. 87.5%) [14]. In melanoma, van der Ploeg et al. used SPECT/CT imaging to investigate the anatomical distribution of ILNs in lower-limb MM. They observed that more than half (51%) of imaging SLNs were located in the inferior medial zone, with positive SLNs predominantly enriched in this region (83%) [15]. These observations motivate further mapping studies but do not validate a reduced dissection template in melanoma. Despite these findings, the comprehensive landscape of ILN distribution in lower-limb MM remains uncertain, with a lack of validation via pathologically positive nodes.

This retrospective study aimed to describe the pathological distribution of ILNs in lower-limb MM using five anatomically defined zones. Zone-based mapping could guide the design of future studies of surgical extent, standardized specimen assessment, and imaging–pathology correlation. The present analysis was not designed to establish a reduced dissection template or to demonstrate its oncological safety.

## 2. Materials and Methods

### 2.1. Study Population

**Figure 1:**
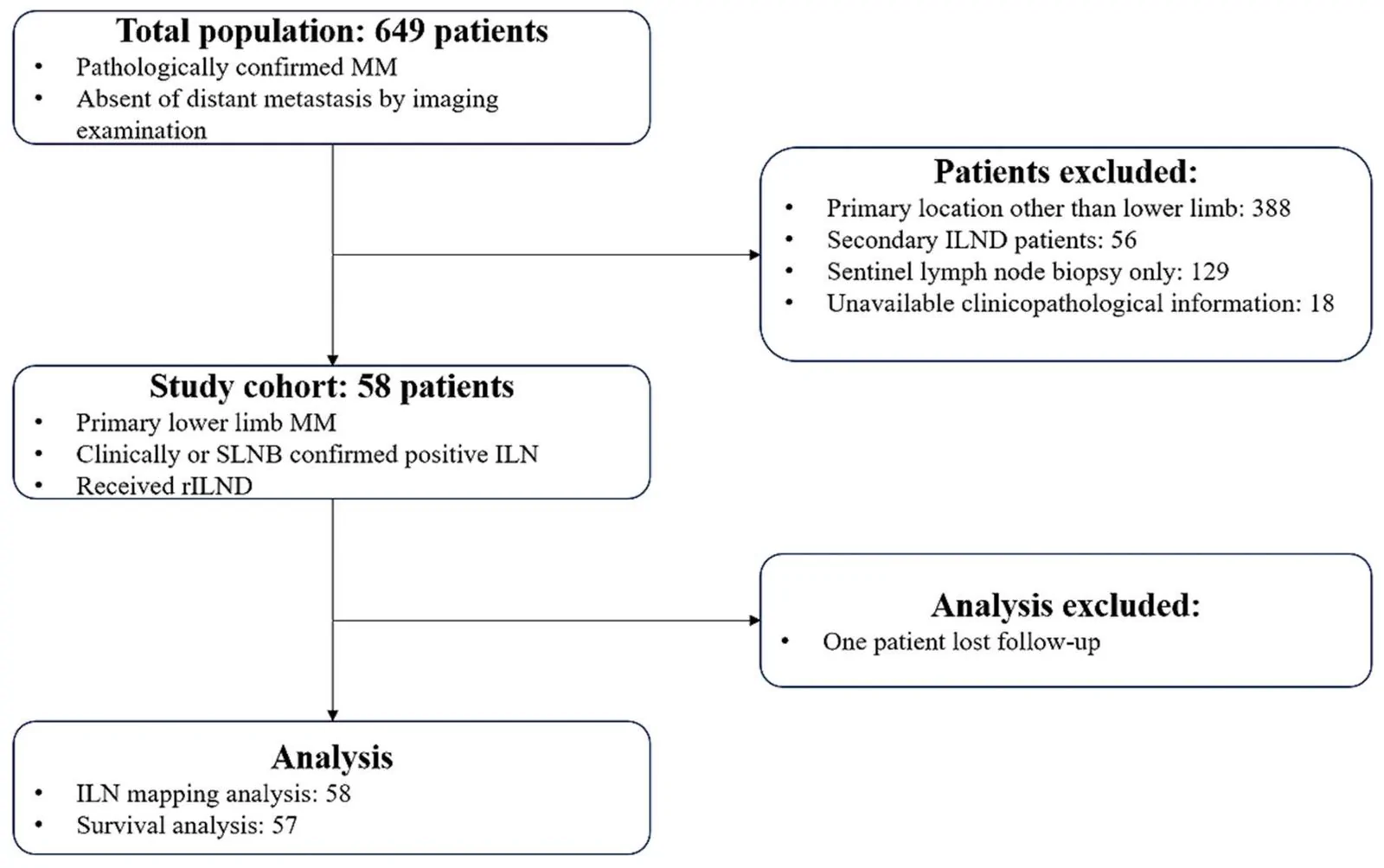
Screening and analysis populations. Of 649 patients in the screening population, 388 were excluded because the primary site was outside the lower limb, 56 because of secondary ILND, 129 because they underwent SLNB only, and 18 because clinicopathological information was unavailable (591 exclusions in total). The pathological mapping cohort comprised 58 patients: 48 with clinically positive ILNs and 10 with positive SLNB. The original survival analysis included 57 patients and excluded one patient described as lost to follow-up. MM: malignant melanoma; SLNB: sentinel lymph node biopsy; ILN: inguinal lymph node; ILND: inguinal lymph node dissection; rILND: radical inguinal lymph node dissection.

From March 2018 to November 2024, 649 patients with MM were pathologically diagnosed at Peking University Cancer Hospital. Patients with distant metastasis were excluded and recommended for systemic therapy. SLNB was performed for patients with clinically negative nodal basins, as described in a previous study [16]. SLNB-positive patients were offered active nodal basin surveillance or CLND. During the study period, patients with clinically positive ILNs were recommended to undergo therapeutic rILND. This study included patients with lower-limb MM who underwent rILND. The screening process and exclusion counts are shown in Figure 1. The analysis combined clinically node-positive patients and SLNB-positive patients; these distinct clinical settings were considered when interpreting the results. Patients with three or more positive ILNs, positive Cloquet’s nodes, or clinically positive pelvic lymph nodes underwent additional pelvic lymph node dissection. All patients provided informed consent, and the protocol was approved by the institutional ethics committee. The study adhered to the Declaration of Helsinki. Pelvic nodal status was recorded as confirmed positive after dissection versus a combined category of negative or not dissected. Because a separate dissection-status indicator was unavailable in this dataset, pelvic findings were summarized descriptively without treating the combined category as pathologically negative.

### 2.2. Surgical Boundaries and Anatomical Division of ILNs

**Figure 2:**
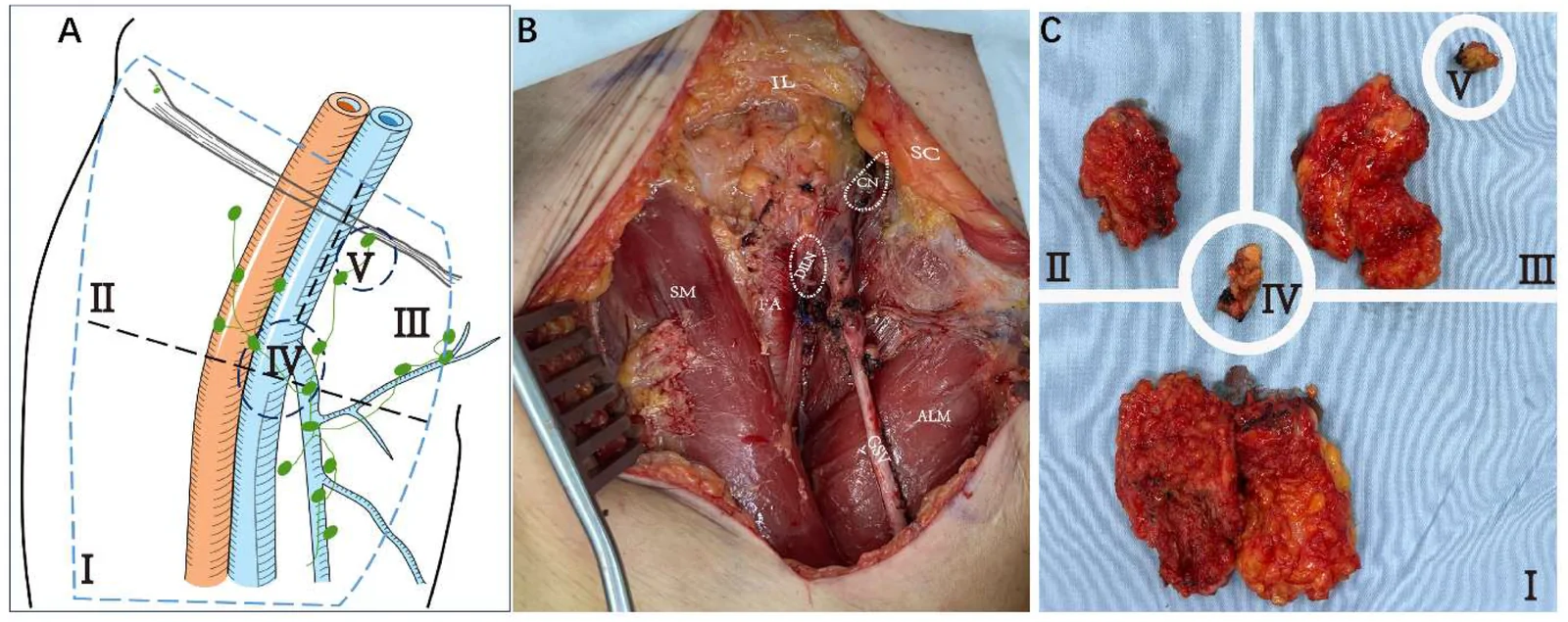
Surgical boundaries and anatomical zones. (**A**) The blue outline indicates the radical inguinal lymph node dissection boundary. A line through the saphenofemoral junction, parallel to the inguinal ligament, separates inferior superficial zone I from superior superficial zones II and III. Above this line, zone II lies lateral and zone III medial to the femoral vein. Zone IV comprises deep ILNs medial to the femoral vessels and deep to the fascia lata; zone V comprises Cloquet’s nodes medial to the femoral vein beneath the inguinal ligament. (**B**) Operative field after removal of deep ILNs and Cloquet’s node. (**C**) Separately submitted specimens from the five zones. IL: inguinal ligament; SC: spermatic cord; ALM: adductor longus muscle; SM: sartorius muscle; GSV: great saphenous vein; FA: femoral artery; CN: Cloquet’s node; DILN: deep inguinal lymph node.

The surgical boundaries of rILND are shown in Figure 2A,B. The dissection was defined by the following anatomical landmarks: medial margin was adductor longus; lateral margin was sartorius; the superior margin was 2 cm above the inguinal ligament; the inferior margin was the distal point of the femoral triangle. Superficial ILNs were removed entirely and categorized into three zones based on anatomical landmarks (saphenofemoral intersection and femoral vein, Figure 2C). Zone I was defined as the superficial ILNs located below a line parallel to the inguinal ligament that passes through the saphenofemoral junction. Zones II and III were defined as the superficial ILNs located above the saphenofemoral junction; zone II was lateral to the femoral vein, and zone III was medial to it. The deep ILNs were primarily located along the medial aspect of the femoral vessels, deep to the fascia lata, and removed separately as zone IV. Cloquet’s nodes were located deep to inguinal ligament and medial to femoral vein, removed separately and designated as zone V.

### 2.3. Pathological Examination

The primary tumors and dissected zones were submitted separately for pathological analysis. Pathologists, blinded to the anatomical origins of the nodes, processed the lymph node basins using a standard protocol. This included isolating nodes from surrounding adipose tissue, embedding them in paraffin, and sectioning at 5 mm intervals. Paraffin-embedded specimens were examined by light microscopy at magnifications of ×40 and ×200. Additional immunohistochemical (IHC) staining (S100, HMB-45 and Melan-A) was used for suspicious cases identified with H&E. Additional serial sectioning (at a 2 mm interval) and IHC was performed on all doubtful cases to detect micrometastasis. Extranodal extension (ENE) was defined as tumor penetration beyond the lymph node capsule or metastatic tumor presence in extranodal tissue. The number of positive nodes and total nodes in each zone were recorded. Tumors and regional lymph nodes were staged according to the 8th edition of the American Joint Committee on Cancer (AJCC) TNM system.

### 2.4. Follow-Up and Survival

Postoperative patients were advised to undergo systemic adjuvant therapy. Imaging was conducted every 3 months for the first 2 years, then every 6–12 months for the following 3 years. Disease-free survival (DFS) was defined as the interval from surgery to tumor recurrence or death. Overall survival (OS) was defined as the interval from surgery to death from any cause.

### 2.5. Statistical Analysis

Node-level summaries were calculated from the five zone-specific positive and examined counts. The mapping was inferior = zone I, superolateral = zone II, superomedial = zone III, deep group = zone IV, and femoral canal/Cloquet’s = zone V. A within-zone positivity rate was the number of positive nodes divided by all examined nodes in that zone; the share of all positive nodes used the total positive-node count across zones as its denominator. Zero examined nodes indicated no node available for pathological assessment in that zone, not confirmed absence of disease. Patient-level total positive-node count and number of involved zones were derived directly from these counts. Multiple-zone involvement was defined as ≥2 zones with at least one positive node. Five patients with no positive mapped nodes were retained in the overall 58-patient analysis but excluded from positive-node distribution subgroups. Node-level rates and surgical-indication subgroup comparisons were descriptive; no independent-node significance tests were applied. Exploratory logistic regression reported odds ratios (ORs), Wald 95% confidence intervals (CIs), and two-sided *p* values. Univariable analyses evaluated ≥3 positive ILNs and extranodal extension (ENE). A parsimonious, revision-stage multivariable model included these two factors without stepwise selection. Uncertain, unreported, or missing ENE was treated as missing (8 patients), leaving 50 complete cases and 18 multiple-zone events for the adjusted model. A sensitivity analysis excluded patients with no positive mapped nodes. These models were not externally validated. Kaplan–Meier survival estimates are retained as descriptive, unadjusted results. The nodal reanalysis was performed using Python (version 3.12.14) with NumPy (version 2.3.5), pandas (version 2.2.3), and SciPy (version 1.17.0).; logistic maximum-likelihood estimates used the observed information matrix for standard errors.

## 3. Results

### 3.1. Baseline Characteristics of 58 Lower-Limb MM Patients Receiving rILND

A total of 58 lower-limb MM patients underwent rILND, including 48 (82.8%) with clinically positive ILNs and 10 (17.2%) with positive SLNB (Table 1). The primary lesions were predominantly acral (47/58, 81.0%). The mapped zone records contained no positive ILNs in five patients: three from the clinically node-positive group and two from the SLNB-positive group. Among the SLNB-positive patients, the manuscript imaging record localized SLNs to zone I in 8/10 cases. Nine patients (15.5% of the full cohort) had recorded pathologically positive pelvic nodes. Their inguinal positive-node counts ranged from 1 to 11 (median, 4); five had ≥3 positive ILNs, four had multiple-zone involvement, and three had positive zone V nodes. Of the other six pelvic-positive patients, two had examined but negative zone V nodes and four had no zone V node retrieved. Postoperatively, the study records 49 patients receiving PD-1 monotherapy, six receiving BRAF inhibitors, and three declining adjuvant therapy. The previously reported median follow-up, DFS, and OS were 15.8, 6.8, and 22.7 months, respectively; these survival estimates were not re-estimated in the nodal reanalysis.

### 3.2. Pathological Distribution of ILNs

**Figure 3:**
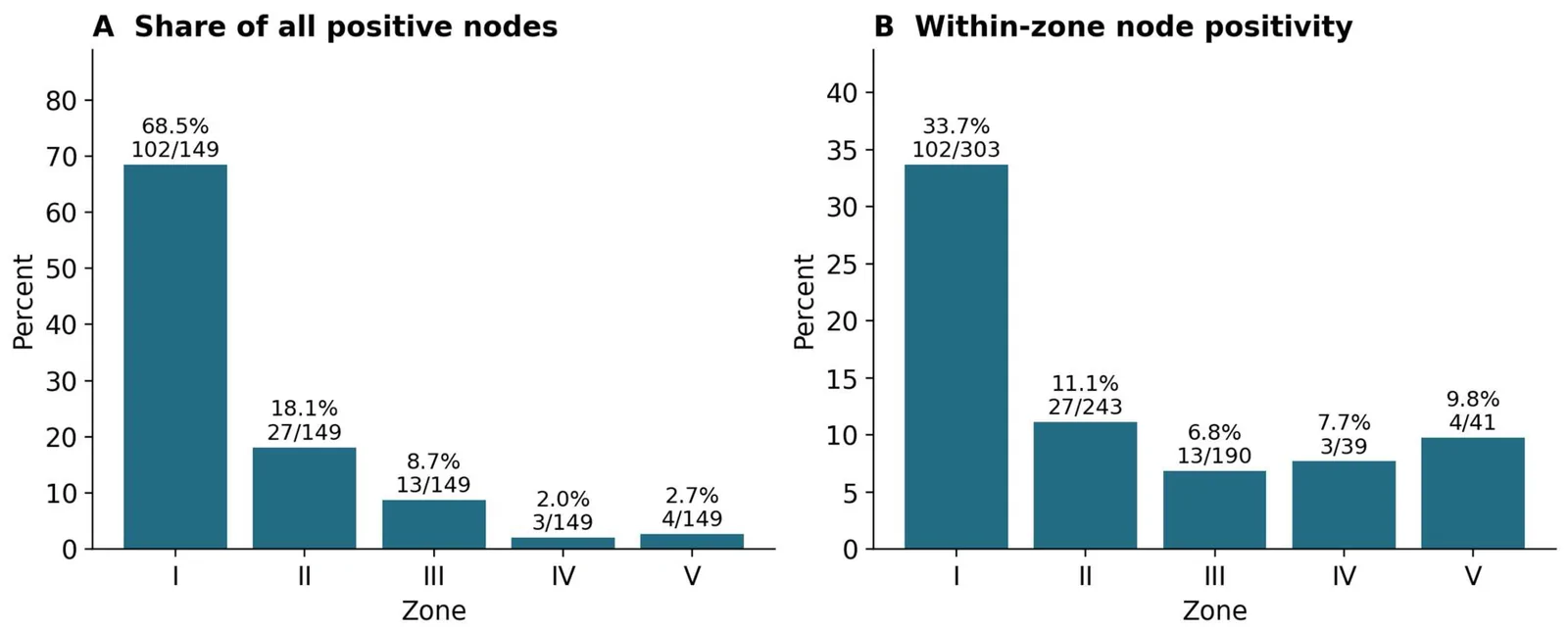
Node-level distribution and positivity. (**A**) Share of all 149 positive mapped nodes contributed by each zone. (**B**) Positive nodes divided by all examined nodes within each zone. Labels show exact numerators and denominators. These are descriptive summaries and do not treat individual nodes as independent observations for hypothesis testing.

The 58 patients had 816 mapped ILNs, including 149 positive nodes; the mean nodal yield was 14.07 ± 4.56 per patient. Zone I contained 102/149 positive nodes (68.5%), followed by zone II (27/149, 18.1%), zone III (13/149, 8.7%), zone V (4/149, 2.7%), and zone IV (3/149, 2.0%). Within-zone positivity rates were 102/303 (33.7%), 27/243 (11.1%), 13/190 (6.8%), 3/39 (7.7%), and 4/41 (9.8%) for zones I–V, respectively (Table 2 and Figure 3). At least one node was retrieved from zones I, II, III, IV, and V in 58, 58, 55, 26, and 27 patients, respectively. The low yield in the deep compartments limits interpretation of apparent non-involvement. In the clinically node-positive group, zone I contained 81/118 positive nodes (68.6%) and its within-zone positivity rate was 81/267 (30.3%); corresponding values in the SLNB-positive group were 21/31 (67.7%) and 21/36 (58.3%). Multiple-zone involvement occurred in 16/48 and 4/10 patients, respectively. These small, selected groups should not be interpreted as having equivalent distributions.

**Figure 4:**
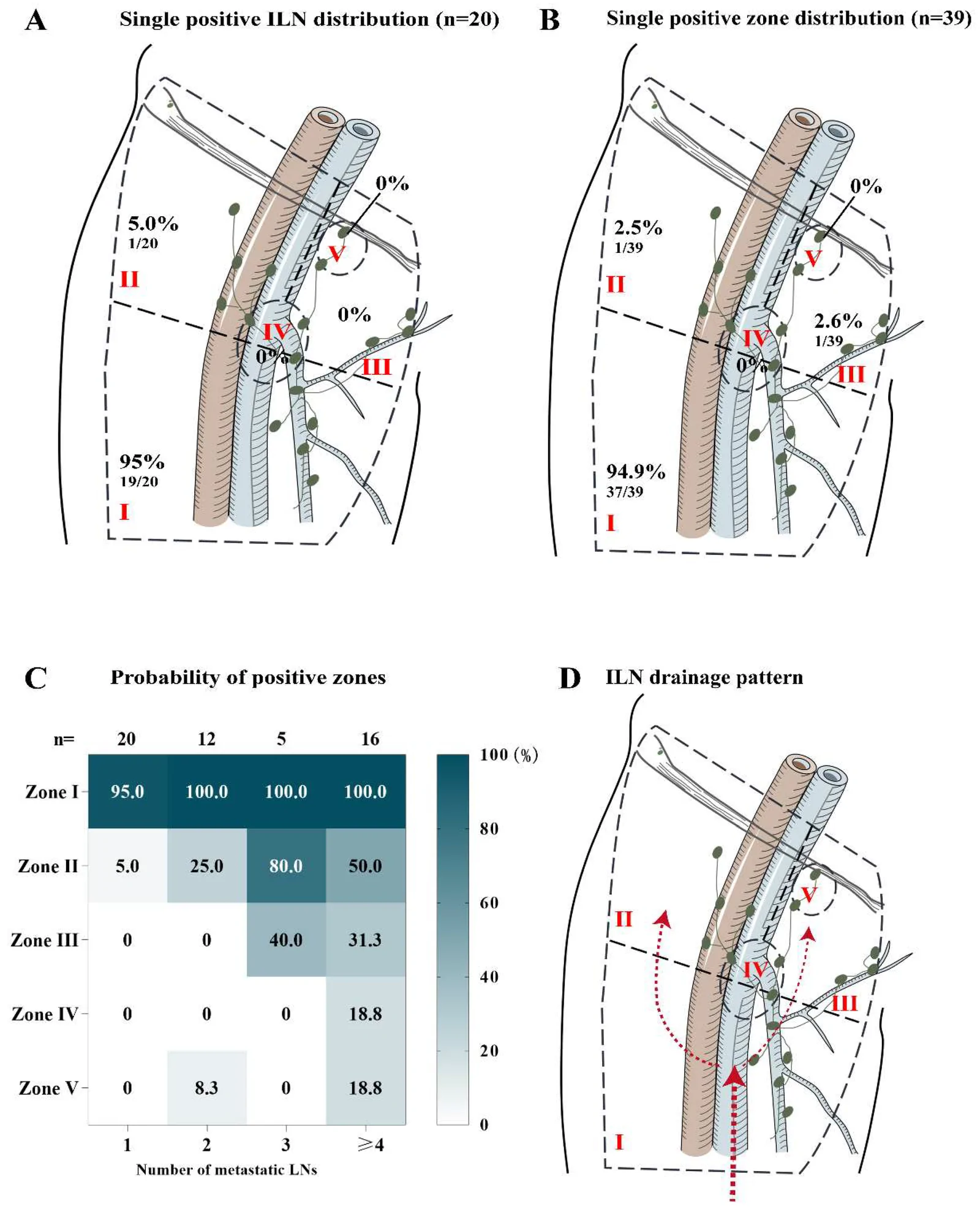
Distribution of first-tier positive ILNs and lymphatic drainage pattern. (**A**). Percentage of single positive ILNs across different zones. (**B**). Proportion of first-tier metastatic zones among patients with single-zone positivity. (**C**). Heatmap illustrating the likelihood of zone involvement when multiple positive ILNs are present. (Rows represent anatomical zones; columns represent patients with 1, 2, 3, or ≥4 positive ILNs. The column denominators are 20, 12, 5, and 16 patients. Each cell equals the number of patients with ≥1 positive ILN in that zone divided by the number of patients in that column, multiplied by 100. Color intensity indicates the observed percentage, not a predicted probability of future spread. Multiple zones may be involved in the same patient.) (**D**) Proposed lymphatic drainage pattern of ILNs in lower-limb MM. Red lines and arrows indicate drainage pathways, with thicker lines representing higher probabilities of lymphatic flow. ILN: inguinal lymph node. LN: lymph node.

An earlier pathological mapping study in penile cancer used the location of a single positive ILN to describe metastatic distribution [17]. In the present cohort, 19/20 patients with a single positive ILN had that node in zone I (95.0%); the remaining node was in superomedial zone III (Figure 4A). Thirty-three patients had positive nodes confined to one anatomical zone: 32/33 (97.0%) in zone I and 1/33 (3.0%) in zone III (Figure 4B). Twenty patients had ≥2 involved zones. Single-node and single-zone involvement describe pathological findings at surgery and are not equivalent to identification of a sentinel node or proof of the first site of metastatic spread.

Figure 4C describes the 53 patients with at least one positive mapped ILN; the five patients with zero positive mapped nodes are not included in this positive-node stratification. The groups with 1, 2, 3, and ≥4 positive ILNs contained 20, 12, 5, and 16 patients, respectively. For example, zone II was involved in 0/20 (0%), 3/12 (25.0%), 4/5 (80.0%), and 8/16 (50.0%) patients, respectively. Percentages across zones need not sum to 100% because a patient may have multiple involved zones. These cross-sectional findings suggest an association between nodal burden and anatomical extent, but do not establish a temporal sequence of spread.

### 3.3. Factors Associated with Multiple-Zone Involvement

**Figure 5:**
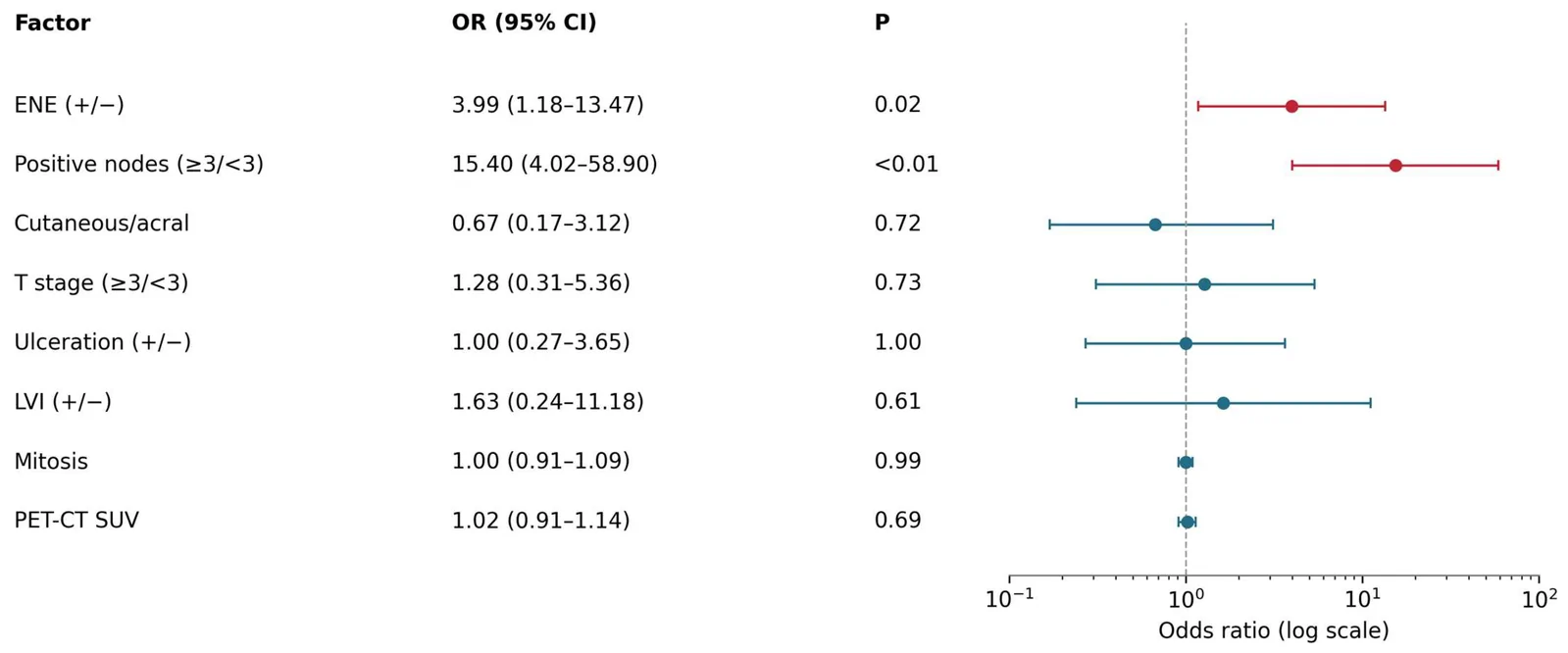
Forest plot of univariable odds ratios for multiple–zone involvement from binary logistic regression. Points indicate odds ratios and horizontal lines indicate 95% confidence intervals. ENE: extranodal extension; LVI: lymphovascular invasion; SUV: standardized uptake value; OR: odds ratio; CI: confidence interval.

In univariable binary logistic regression, ENE (odds ratio [OR], 3.99; 95% confidence interval [CI], 1.18–13.47) and ≥3 positive ILNs (OR, 15.40; 95% CI, 4.02–58.90) were associated with multiple-zone involvement (Figure 5). These are cross-sectional associations rather than time-to-event effects or validated preoperative predictors. The reported multivariable analysis identified ≥3 positive ILNs as statistically significant.

## 4. Discussion

This selected cohort comprised predominantly clinically node-positive and acral lower-limb melanomas. Pathological mapping showed that zone I contained the largest share of positive ILNs, including 19/20 single-positive nodes and 32/33 cases with single-zone involvement. Other zones were more frequently involved in strata with a higher nodal burden, and ENE and ≥3 positive ILNs were associated with multiple-zone involvement in univariable analyses. Together, these findings support preferential inferior-zone involvement, while providing no direct evidence of sequential spread or the safety of a smaller dissection.

Building on Daseler’s five-zone classification, which lacks clear anatomical landmarks, we proposed a modified method for lower-limb MM. In our approach, we redefined the superficial ILNs into zone I-III using precise anatomical landmarks (the saphenofemoral intersection and femoral vein), eliminated the ambiguous central zone, and emphasized the inclusion of indispensable deep ILNs (zone IV: medial to the femoral vessels, deep to the fascia lata) and Cloquet’s node (zone V: medial to the femoral vein, deep to the inguinal ligament).

Zone I accounted for 68.5% of all positive ILNs and had the highest observed within-zone positivity rate (33.7%, compared with 6.8–11.1% in the other zones). These are distinct descriptive measures: one characterizes the location of positive nodes, while the other uses the examined yield within each zone as its denominator. Neither measure proves that other zones can be omitted safely. The findings are consistent with preferential inferior drainage reported by van der Ploeg et al., who found 83% of positive sentinel nodes in the inferior medial region [15]. However, the anatomical templates and clinical populations differ. The 20-patient single-node subgroup, 33-patient single-zone subgroup, and 8/10 SLNB localization finding require external validation. Of particular relevance, node retrieval was recorded in only 26 and 27 patients in zones IV and V, respectively, so a zero count cannot uniformly be interpreted as a negative pathological assessment.

Differences between melanoma and penile cancer may reflect primary-site anatomy and tumor biology. Penile lymphatics include superficial and deep drainage systems [18], whereas cadaveric studies describe lower-limb pathways converging on inguinal nodes [19]. Such anatomy provides a plausible explanation for preferential inferior involvement, but does not demonstrate obligatory stepwise progression in melanoma. At a molecular level, a human melanoma study associated primary-tumor VEGF-C expression and increased peritumoral lymphatic vessel area with SLNB positivity [20]. This supports a possible role for tumor-associated lymphangiogenesis in nodal dissemination. VEGF-C, lymphatic vessel density, and related pathways were not measured here, and cannot explain zone-specific involvement on the basis of these data.

ENE and ≥3 positive ILNs were associated with multiple-zone involvement in univariable analyses; only ≥3 positive ILNs retained statistical significance in the two-variable adjusted model. Previous melanoma studies linked sentinel-node tumor burden and ENE with additional non-sentinel-node disease and adverse outcomes [21,22]. However, both ENE and the total number of positive nodes are primarily postoperative findings, and cannot serve as established preoperative eligibility criteria for reduced rILND. Moreover, total positive-node count is structurally related to the opportunity for involvement of multiple zones. Candidate assessments for future validation include nodal ultrasound, cross-sectional imaging, image-guided biopsy, and, where clinically indicated, sentinel-node mapping. A negative imaging or intraoperative assessment cannot be assumed to exclude microscopic disease in an omitted zone.

Contemporary management also changes the population selected for dissection. MSLT-II and DeCOG-SLT support surveillance after a positive SLNB rather than routine CLND [4,5], while recent European guidance includes neoadjuvant treatment for selected patients with clinically apparent, resectable nodal disease [6,7]. An Italian multi-center study of 4938 newly diagnosed melanomas reported persistently thicker tumors, more ulceration, more stage II–III disease, and greater SLNB positivity during and after the COVID-19 pandemic compared with the pre-pandemic period [23]. These findings illustrate how diagnosis period and referral patterns can change case mix; they cannot establish the same temporal trend in our predominantly acral Chinese cohort.

Reducing morbidity remains a relevant surgical aim, particularly because groin dissection disrupts lymphatic channels and creates a substantial wound and dead space. The melanoma-specific literature describes wound complications and lymphedema after inguinal dissection [8,9,10,11]. Cananzi et al. reported an inguinal “neoligament” reconstruction technique after groin soft tissue sarcoma resection [24], providing adjacent surgical context for complex groin reconstruction. Sarcoma resection differs from melanoma lymphadenectomy, and this report does not establish melanoma-specific complication rates or support reduced rILND. Any proposed reduction in surgical extent must be evaluated against the risks of residual disease and nodal-basin recurrence.

### 4.1. Strengths and Limitations of the Study

A strength of this study is the anatomically defined, zone-based pathological assessment after rILND, including separate evaluation of deep and Cloquet’s nodes. The analysis distinguishes single-node from single-zone involvement and provides a framework for standardized pathological mapping. However, the retrospective single-center design and small sample limit precision and generalizability. The cohort was enriched for clinically positive ILNs (48/58, 82.8%), acral primary sites (81.0%), and advanced primary tumors as described in the original clinical records. Selection for surgery, surgeon-related differences in dissection and specimen allocation, and variation in nodal yield and tumor burden may have influenced the observed distribution. The findings cannot be generalized directly to all SLNB-positive patients or those managed by surveillance.

The pooled cohort combines therapeutic dissection for clinically evident disease and CLND after positive SLNB, which represent different nodal burdens and treatment settings. The SLNB subgroup comprised only 10 patients, and the single-positive-node subgroup only 20. In addition, pathological mapping at one time point cannot establish the order of metastatic spread, and previous evidence from penile cancer cannot validate melanoma-specific reduced dissection boundaries. Possible differences in pathological sampling and micrometastasis detection may also affect zone assignment and sensitivity. The reason for zero positive mapped nodes in five patients, including two with positive SLNB, cannot be established from the zone counts alone; complete reconciliation with earlier SLNB specimens would be required to establish cumulative basin burden. Systematic preoperative systemic-treatment data were unavailable for analysis, so treatment-related changes in nodal pathology could not be evaluated.

The regression results are exploratory and vulnerable to sparse events and residual confounding. The adjusted model included only positive-node burden and ENE, not a comprehensive set of primary thickness, ulceration, T stage, clinical nodal status, and examined-node count. ENE-containing models excluded eight uncertain or missing records, and the wide CIs indicate limited precision. Positive-node count is structurally related to the opportunity for involvement of multiple zones, limiting causal and predictive interpretation. The pelvic field combined negative and undissected patients; consequently, a pelvic positivity rate among dissected patients and a valid positive-versus-pathologically-negative comparison could not be estimated. Survival estimates remain descriptive because follow-up was short and systemic therapy was not adjusted for. The study did not compare reduced with standard rILND or establish nodal-basin control, morbidity reduction, or safety after a reduced procedure.

### 4.2. Future Directions

Multi-center studies should first validate the five-zone map using standardized surgical landmarks, specimen labeling, and pathology protocols. Clinically node-positive and SLNB-positive patients should be analyzed separately, with explicit recording of neoadjuvant treatment, pathological response, pelvic-node status, and node-level denominators. Prospective imaging–pathology correlation is needed to determine whether preoperative or intraoperative findings can identify patients at low risk of disease in an omitted zone. A reduced dissection should then be evaluated prospectively against standard surgery, with predefined nodal-basin recurrence, disease control, survival, lymphedema, wound complications, and patient-reported outcomes. Postoperative ENE and node counts alone are insufficient to select patients for an initial reduced operation.

## 5. Conclusions

This small, selected single-center retrospective cohort provides preliminary pathological mapping evidence of preferential inferior-zone involvement in lower-limb melanoma. The findings support further investigation of anatomical patterns but do not establish sequential spread, validated selection criteria, or the oncological safety of reduced rILND. Surgical practice should not be changed on these data alone; external and prospective validation are required.

## Figures and Tables

**Table 1:** Baseline characteristics of 58 lower-limb MM patients.

Variables	N (%)
Gender	
Male	28 (48.3)
Female	30 (51.7)
Age (median, range)	60 (26–79)
Primary lesion	
Acral	47 (81.0)
Cutaneous	11 (19.0)
T stage	
Tis	1 (1.7)
1	3 (5.2)
2	6 (10.3)
3	13 (22.4)
4	25 (43.1)
NA	10 (17.2)
Ulceration	
+	33 (56.9)
−	15 (25.9)
NA	10 (17.2)
LVI	
+	5 (8.6)
−	38 (65.5)
NA	15 (25.9)
Mitosis/10 HPF (median, range)	3.0 (1.0–35.0)
Indication of rILND	
SLNB-positive	10 (17.2)
Clinically positive	48 (82.8)
Documented pelvic nodal metastasis	
Positive	9 (15.5)
Negative or not dissected	49 (84.5)

LVI: lymphovascular invasion; HPF: high-power field; rILND: radical inguinal lymph node dissection; NA: not available.

**Table 2:** Zone-specific pathological node counts in 58 patients.

Zone	Positive/Examined Nodes	Within-Zone Positivity	Share of Positive Nodes	Patients with ≥1 Examined Node
I	102/303	33.7%	102/149 (68.5%)	58/58
II	27/243	11.1%	27/149 (18.1%)	58/58
III	13/190	6.8%	13/149 (8.7%)	55/58
IV	3/39	7.7%	3/149 (2.0%)	26/58
V	4/41	9.8%	4/149 (2.7%)	27/58

## Data Availability

The raw data supporting the conclusions of this article will be made available by the authors on request.

## Abbreviations

The following abbreviations are used in this manuscript:

MM	Malignant melanoma
rILND	Radical inguinal lymph node dissection
ILN	Inguinal lymph node
ENE	Extranodal extension
SLNB	Sentinel lymph node biopsy
CLND	Completion lymph node dissection
LN	Lymph node
PC	Penile cancer
DFS	Disease-free survival
OS	Overall survival
OR	Odds ratio
